# Treatment with betablockers is associated with higher grey-scale median in carotid plaques

**DOI:** 10.1186/1471-2261-14-111

**Published:** 2014-08-30

**Authors:** Giuseppe Asciutto, Nuno V Dias, Ana Persson, Jan Nilsson, Isabel Gonçalves

**Affiliations:** Vascular Center Malmö-Lund, Skåne University Hospital, Ruth Lundskogs gata 10, 1st floor, Malmö, 205 02 Sweden; Experimental Cardiovascular Research Unit, Clinical Research Center, Department of Clinical Sciences Malmö, Lund University, Malmö, Sweden; Department of Cardiology, Skåne University Hospital, Malmö, Sweden

**Keywords:** Betablockers, Grey-scale median, Carotid artery, Carotid plaque

## Abstract

**Background:**

The presence of echolucent carotid plaques as defined by low ultrasound grey-scale median (GSM) is associated with a higher risk of stroke and myocardial infarction. Betablockers have shown possible anti-atherosclerotic effects. The aim of the present study was to determine if there is an association between carotid plaque GSM and treatment with betablockers.

**Methods:**

The GSM of the carotid plaques of 350 patients who underwent carotid endarterectomy (CEA) for asymptomatic (n = 113) or symptomatic (n = 237) carotid disease was measured. Patients were divided in two groups based on the absence/presence of an on-going long-term (*i.e.* at least 6 months) oral treatment with betablockers at the time of CEA.

**Results:**

The prevalence and type of preoperative neurological symptoms were similar in the two groups. Patients with betablockers had more frequently arterial hypertension (P < .0001), diabetes (P = .035) and a higher BMI (P = .0004), while patients without betablockers were most frequently smokers (P = .017).

Patients with betablockers revealed to have higher GSM (37.79 ± 25 *vs* 32.61 ± 23.50 P = .036). Echogenic plaques (i.e. with GSM > 30) showed to be more frequent in patients with betablockers also after correction for age, gender, the occurrence of preoperative symptoms, diabetes, hypertension, smoking and statins use (P = .024).

**Conclusions:**

These results suggest the use of standardized ultrasound techniques as an important tool in evaluating the effect of anti-atherosclerotic medications and underline the need of.further prospective randomized studies on larger patient cohorts in order to confirm these results.

## Background

B-mode ultrasound of the carotid arteries has shown to be a valid method for non-invasive assessment of the presence and clinical burden of atherosclerosis [1, 2].

There is today a general consensus that the risk of an atherosclerotic plaque to become symptomatic is not only dependent on the degree of stenosis but also on its composition.

The possibility to assess carotid plaque morphology on ultrasound images in a standardized way has been widely explored in the last decade mostly by the use of the grey-scale median (GSM). Echolucent plaques, which have shown to be rich in lipids and macrophages as expression of a high degree of intrinsic plaque inflammation [3–5].

Treatment with statins has already shown to influence plaque composition and its ultrasound appearance [6, 7]. As perioperative beta-blockade became standard therapy in the prevention of cardiovascular (CV) events in patients undergoing vascular surgery, the question about their mechanisms of action was raised. Several hypotheses such that of a reduced inflammatory response which leads to the atherogenic process [8], or the effects by the promotion of laminar flowpatterns [9] have been advanced.

Furthermore, betablockers have been shown to have an inhibitory effect on early stages of carotid atherosclerosis development [10, 11] and a long-term exposition to betablockershas been associated to a milder degree of inflammation in carotid plaques [12].

The aim of the current study is to investigate the possible influence of long-term treatment with betablockers on the GSM of carotid plaques of patients using it as a surrogate marker of the effects on plaque composition.

## Methods

### Study population

410 patients who underwent carotid endarterectomy (CEA) between October 2005 and December 2012 at our Vascular Department were identified for inclusion in this study. Informed consent was given by all patients and the study was approved by the local ethical committee (Regional ethical committee, Lund - decision number 472/2005).

Sixty patients were excluded because of the presence of extremely calcified plaques not allowing a GSM measurement, accounting for a total of 350 analysed plaques.

Patients with ipsilateral carotid artery occlusion, radiation carotid artery stenosis or restenosis after previous CEA or endovascular treatment were excluded. An independent accredited neurologist clinically assessed all patients preoperatively. Patients were considered to have asymptomatic disease if they had no *amaurosis fugax* (AF), transient ischemic attacks (TIAs) or stroke in the 6 months prior to surgery. Indications for surgery were described in detail elsewhere [13].

Information about comorbidities and past medical history were obtained through standardized preoperative interviews and review of the medical records. Comorbidities such as hypertension, diabetes, smoking, as well as the body mass index (BMI), serum levels of triglycerides (TG), low-density lipoproteins (LDL), high-density lipoproteins (HDL) were recorded.

The Swedish national medication register was analysed to identify and determine the presence of any on-going treatment with betablockers at the time of surgery and its duration. This is a nationwide healthcare database where all the prescriptions are registered. Long-term treatment was assumed in patients receiving betablockers of any type and dose for at least six months before surgery, regardless of the primary indication. All patients undergoing CEA were routinely prescribed long-term treatment with statins (simvastatin 40 mg) and acetylsalicylic acid (75 mg) or clopidogrel (75 mg) daily, if this treatment was not already in use.

### B-mode ultrasound and measurement of GSM

One day before surgery, carotid ultrasound investigation was performed by a certifies sonographer using an Acuson Sequoia (Acuson, Mountain View, CA, USA) with a 6 MHz-probe according to a previously published method [14].

All images for measurement of GSM in the plaque were obtained in the longitudinal projection showing the largest plaque present. GSM analysis was performed on a longitudinal image showing the largest plaque present in a predefined window consisting of three centimeters of the common carotid artery (CCA), the bulb and one centimeter of the internal- and external carotid artery (ICA and ECA respectively).

A video colour flow sequence was recorded in order to help in outlining the plaque for GSM analysis. All GSM measurements were performed by a single specially trained and experienced sonographer (G.A.) with blinded patient information, including current medications. An intravariability study (n = 20) for measurement of GSM showed that the method had good reproducibility, with a coefficient of variation of 7% and absolute difference between measurements was 1.5 units.

Measurement of GSM was done using Adobe Photoshop 12.0 (Adobe System incorporated, San Jose, CA, USA) with a method previously described [15]. In brief, the method is based on the standardization of images where black, represented by lumen, and white, represented by the adventitia, are given the values 0 and 190, respectively. After linear adjustment of the grey scale in the images according to these values, the plaque was outlined. The median value of the grey scale, the GSM value, in this outlined area was used to define the degree of echogenicity in the plaque.

### Statistical analysis

Continuous variables are presented as mean ± standard deviation (SD) when not stated otherwise, while categorical variables are presented as percentages.

As the GSM values of the entire study population were non-normally distributed, its median was used to divide plaques in to echolucent (≤30) and echogenic (>30).

Mann-Whitney U test and Spearman’s rank correlation were used for GSM analysis for the same reason.

Simple and multiple linear regressions were used to explore the relationship between two or more continuous variables, while logistic regression was used in case of dichotomous variables. The regression model included age, gender, hypertension, diabetes, tobacco use, pre-operative symptoms and use of statins. A P-value of < .050 was considered statistically significant. Statistical analysis was performed using SPSS 22.0 (IBM Corp., Amonk, NY: USA).

## Results

The plaques of three-hundred and fifty patients (70 ± 9 years, 245 males) undergoing CEA were included. Two-hundred and thirty seven (68%) plaques were associated with ipsilateral hemispheric symptoms while 113 (32%) were not.

The study population was divided into two subgroups based on the absence (n = 197) or presence (n = 153) of long-term treatment with betablockers at the time of surgery. Nine different types of betablockers were used (metoprolol 72%, atenolol 20%, bisoprolol 4%, others 4%), with a mean time of treatment duration was of 53.2 ± 22.3 months. Overall 87% of the subjects were on statins for at least 1 month before CEA (mean treatment time 46.5 ± 23 months).

As shown in Table 1, patients with betablockers had more frequently arterial hypertension (P < .0001), diabetes (P = .035) and a higher BMI (P = .0004), while patients without betablockers were most frequently smokers (P = .017).

**Table 1:** **Demographics**

	No beta-blockers (n = 197)	Beta-blockers (n = 153)	P
**Age (years)**	69.91 ± 8.79	70.79 ± 8.43	.364†
**Male**	69% (137)	71% (108)	.906*
**BMI**	25.9 ± 3.3	27 ± 3.6	.0004†
**Diabetes**	20% (40)	30% (46)	.035*
**Hypertension**	58% (115)	89% (137)	< .0001*
**Smoking**			.017*
**No**	18% (35)	22% (33)	
**Current**	37% (73)	23% (35)	
**Ex**	45% (89)	56% (85)	
**Statins**	85% (167)	89% (136)	.274*
**Degree of stenosis (%)**	85.6 ± 11.8	84.6 ± 12.2	.320^†^
**Symptoms**			
**Type of symptoms**	72% (141)	63% (96)	.618^†^
**AF**	20% (28)	19% (18)	
**TIA**	35% (50)	42% (40)	
**Stroke**	45% (63)	40% (38)	
**Time to surgery – days**	25 ± 32.4	21.8 ± 22.6	.945^†^
**GSM**	32.6 ± 23.5	37.8 ± 25	.036^†^

Patients are grouped based on the use of beta-blockers.

Categorical variables are presented as percentages with absolute numbers between parenthesis. Continuous variables are presented as mean (standard deviation).

AF = *amaurosis fugax*; BMI = Body mass index; TIA = transient ischemic attack; Time to surgery = time between symptoms (if present) and operation. * = Pearson’s Chi-square; ^†^ = Mann-Whitney U test

Age, the degree of stenosis, the incidence and type of preoperative neurological symptoms, the time between symptoms and CEA as well as the serum levels of TG, LDL, HDL were not different between the two groups of patients.

**Figure 1:**
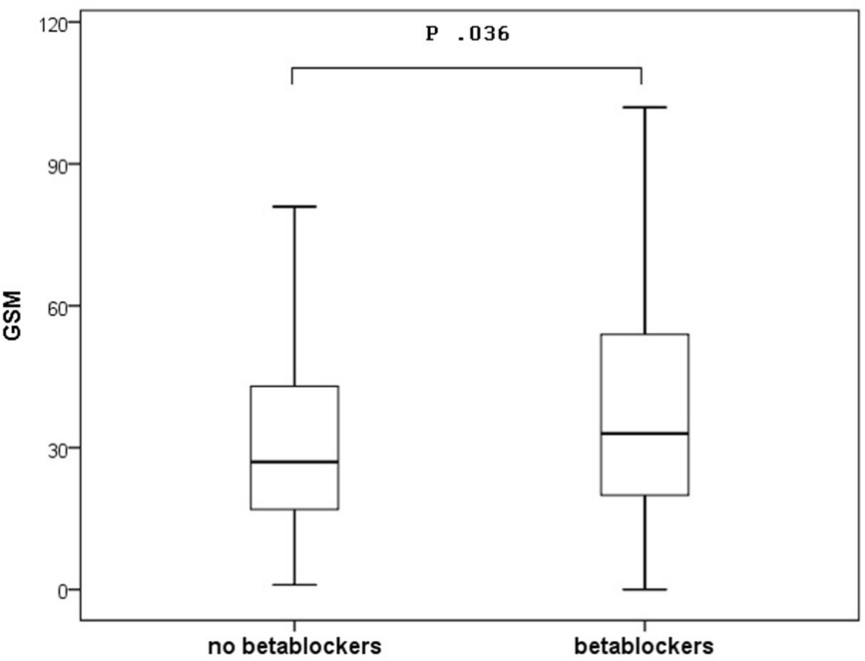
**Box-plots showing the GSM values according to the absence or presence of a long-term treatment with betablockers at the time of surgery.**

As shown in Figure 1, patients with betablockers revealed to have higher GSM than those without (37.79 ± 25 *vs* 32.61 ± 23.50 P = .036).

Echogenic plaques (i.e. with GSM > 30) were more frequent in patients with betablockers . This finding could be confirmed after regression analysis using age, gender, the occurrence of preoperative symptoms, diabetes, hypertension, smoking, BMI and statins use as confounding factors (see Tables 2, 3, 4, 5).

**Table 2:** **Simple linear regression analysis**

	B	St. error	Beta	t	P-value
**Age**	.174	.150	.062	1.157	.248
**Gender**	3.114	2.830	.059	1.100	.272
**BMI**	-.291	.373	-.042	-.779	-.437
**Preoperative symptoms**	-1.070	2.778	-.021	-.385	.700
**Diabetes**	3.878	3.011	.069	1.288	.199
**Hypertension**	2.138	2.869	.040	.745	.457
**Smoking**					
No + Yes 0/Ex 1	-1.299	2.598	-.027	-.500	.617
No + Ex 0/Yes 1	2.561	2.810	.049	.912	.363
**Statins**	-1.690	3.810	-.024	-.444	.658
**Betablockers**	5.177	2.605	.106	1.987	.048

GSM as dependent continuous variable.

**Table 3:** **Multiple linear regression analysis**

	B	St. error	Beta	t	P-value
*Constant*	16.698	19.970		.840	.401
**Age**	.249	.174	.089	1.430	.154
**Gender**	2.830	2.907	.054	.974	.331
**BMI**	-.333	.399	-.048	-.833	.405
**Preoperative symptoms**	-1.693	2.928	-.033	-.578	.563
**Diabetes**	3.641	3.108	.065	1.171	.242
**Hypertension**	-.707	3.125	-.013	-.226	.821
**Smoking**					
No + Yes 0/Ex 1	1.592	3.565	.033	.447	.655
No + Ex 0/Yes 1	5.810	4.127	.111	1.408	.160
**Statins**	-1.496	3.872	-.021	-.386	.699
**Betablockers**	5.786	2.833	.118	2.042	.042

GSM as dependent continuous variable.

**Table 4:** **Simple logistic regression analysis**

	B	St. error	Wald	P-value	Exp(B)	CI for Exp(B)	
						Lower bound	Upper bound
**Age**	.019	.013	2.380	.123	1.019	.995	1.045
**Gender**	.568	.237	5.718	.017	1.765	1.108	2.811
**BMI**	-.031	.031	.984	.321	.970	.913	1.030
**Preoperative symptoms**	.041	.229	.033	.856	1.042	.666	1.632
**Diabetes**	.311	.250	1.547	.214	1.365	.836	2.229
**Hypertension**	.341	.237	2.067	.150	1.407	.883	2.240
**Smoking**							
No + Yes 0/Ex 1	-.413	-.215	3.682	.055	.662	.434	1.009
No + Ex 0/Yes 1	.365	.233	2.443	.118	1.440	.912	2.275
**Statins**	-.194	.315	.378	.539	.824	.444	1.528
**Betablockers**	.550	.218	6.375	.012	1.734	1.131	2.657

GSM ≤ 30 *vs* > 30 as dependent categorical variable.

**Table 5:** **Multiple logistic regression analysis**

	B	St. error	Wald	P-value	Exp(B)	CI for Exp(B)	
						Lower bound	Upper bound
*Constant*	-.874	1.738	.253	.615	.417		
**Age**	.025	.015	2.785	.095	1.025	.996	1.056
**Gender**	.508	.250	4.131	.042	1.662	1.018	2.712
**BMI**	-.029	.035	.698	.403	.972	.908	1.040
**Preoperative symptoms**	-.014	.250	.003	.956	.986	.604	1.610
**Diabetes**	.291	.266	1.192	.275	1.338	.793	2.255
**Hypertension**	.071	.267	.071	.790	1.074	.636	1.813
**Smoking**							
No + Yes 0/Ex 1	-.204	.303	.454	.500	.815	.450	1.476
No + Ex 0/Yes 1	.488	.355	1.886	.170	1.629	.812	3.267
**Statins**	-.202	.330	.373	.541	.817	.428	1.561
**Betablockers**	-.643	.244	6.926	.008	.526	.325	.849

GSM ≤ 30 *vs* > 30 as dependent categorical variable.

## Discussion

The main finding of this study was that long-term treatment with betablockers was associated with higher GSM values of carotid plaques. The degree of stenosis and plaque morphology, defined by its structure and surface characteristics, are thought to play an important role in the pathogenesis of CV events. An unstable carotid plaque is associated with a thinning of the fibrous cap, infiltration of inflammatory cells leading to surface ulceration, and plaque rupture [16, 17]. An early identification of these features suggesting plaque instability would therefore be very useful for treatment. Recent ultrasound studies based on a computerized measurement of the GSM value of the carotid plaque demonstrated that a low GSM value, reflecting echolucent lesions, was associated with an increased risk of cerebrovascular events and may represent a good predictor of carotid plaque behavior [18], leading to suggest GSM measurement as a complementary tool in the planning of the treatment of carotid artery stenosis in addition to neurological symptoms and the degree of stenosis [19].

The possible anti-atherosclerotic effects of several drugs have been investigated. Statins are probably one of the most studied with its use leading to increased GSM both in carotid and coronary plaques [20, 21]. This could be an expression of a more stable plaque phenotype and can be used to monitor their effects on atherosclerotic lesions. Betablockers have been increasingly used in vascular surgery because of their suggested effect in reducing the occurrence of postoperative CV events as suggested by several treatment guidelines [22, 23]. However their indiscriminate use in surgical patients, independently of their CV risk, has been strongly questioned [24]. Even if treatment with betablockers has shown to be associated with possible anti-atherosclerotic effects on early stages of carotid lesion development [10, 11], their mechanisms of action are still unknown. Their antiatherogenic effects could be the result of the reduced endothelial injury related to a decrease in heart rate and pulse pressure, which produce an increase in shear stress and a decrease in pressure related cycling stretching of the artery [25]. Hampering the sympathetic nervous system could also contribute to the antiatherogenic effects of betablockers preventing endothelial injury and reducing the vessel wall permeability to LDL particles [26]. This results in a milder degree of lipid accumulation that, in its turn, produces a more echogenic plaque pattern on ultrasound [3, 27].

A higher degree of intra-plaque inflammation as expressed by a higher content of macrophages [4], and pro-inflammatory cytokines [28] has been shown to lie behind the echolucent aspects of carotid plaques. A possible intrinsic anti-inflammatory action of betablockers, as supported by the results of an animal study [29], could also explain our findings of higher GSM, i.e. more echogenic plaques in patients on long-term treatment with betablockers.

Several lines of evidence suggest that this could possibly reflect a positive effect on atherosclerotic lesions, which, in turn, are associated with a lower risk for development of clinical events [18, 30]. To the best of our knowledge, this is the first study reporting an ultrasound verification of a stabilising effect of the long-term treatment with betablockers on advanced atherosclerotic disease.

As previously reported [12], we could confirm in a larger cohort of patients that those on long-term treatment with betablockers revealed to have more often arterial hypertension, diabetes and a higher BMI. This could suggest a different phenotype than those without, which could be representative of a metabolic syndrome.

A wide range of GSM threshold values, going from 25 to 74, are proposed in the literature to distinguish echolucent from echogenic plaques [18, 19, 28]. These various cut-off points may be attributable to different choices for the standardization of the reference values (lumen and adventitia). For the purpose of the present study, we decided to use the median GSM value of the entire study cohort as cutoff value (i.e. 30).

Moreover, GSM analysis represents a median value of the whole atherosclerotic area and it may not necessarily reflect the presence of particular regional components. New techniques, such stratified analysis [31] and ultrasound risk scores [32] have been developed in order to solve this problem, but their application on large scale have been limited by their complexity so far.

The different therapeutic regimes in terms of betablocker type, absolute dose and duration are bringing heterogeneity to the study population, but tend to reflect clinical practice. Furthermore, the small sample size and the predominant use of metoprolol do not allow any meaningful analysis of the possible influence of the drug type on GSM.

Other simultaneous medical treatments, such as statins and immunotherapy [33], as well as the presence of known and unknown comorbidities could have influenced our findings. The association between long-term treatment with betablockers and higher GSM was, nevertheless, confirmed after statistical correction for statins use and the presence of a known hypertension and diabetes. However, the absence of accurate data on blood pressure and HbA1c can be seen as a limitation of this study.

Its non-randomized design is to see as a limitation of this study and should be overcame in order to be able to make general recommendation about the use of a long-term betablockers treatment in patients with advanced atherosclerotic lesions. Furthermore, the retrospective nature of the study with the absence of a GSM measurement upon the initiation of the betablockers treatment does not allow any kind of speculation about their mechanism of action or their possible direct influence on plaque composition. Prospective, randomized studies, above all on patients with asymptomatic carotid disease are therefore required for confirmatory purposes.

### Conclusions

The findings of the current study support the use of standardized ultrasound techniques as a means of evaluating the effect of anti-atherosclerotic medications. Moreover, our conclusions suggest other effects of betablockers in treating CV diseases beyond hemodynamics. Further studies are needed in order to confirm these results.
